# Benzyldimethyldodecyl Ammonium Chloride Doped Dental Adhesive: Impact on Core’s Properties, Biosafety, and Antibacterial/Bonding Performance after Aging

**DOI:** 10.3390/jfb13040190

**Published:** 2022-10-17

**Authors:** Lamia Sami Mokeem, Abdulrahman A. Balhaddad, Isadora Martini Garcia, Fabrício Mezzomo Collares, Mary Anne S. Melo

**Affiliations:** 1 Ph.D. Program in Biomedical Sciences, University of Maryland School of Dentistry, Baltimore, MD 21201, USA; 2 Department of Restorative Dental Sciences, College of Dentistry, Imam Abdulrahman Bin Faisal University, P.O. Box 1982, Dammam 31441, Saudi Arabia; 3 Dental Materials Department, School of Dentistry, Federal University of Rio Grande do Sul, Porto Alegre 90035-004, RS, Brazil; 4 Operative Dentistry Division, General Dentistry Department, University of Maryland School of Dentistry, Baltimore, MD 21201, USA

**Keywords:** antibacterial agents, dental adhesives, dental caries, quaternary ammonium compounds, polymerization, oral biofilm

## Abstract

Current dental adhesives lack antibacterial properties. This study aimed to explore the effect of incorporating benzyldimethyldodecyl ammonium chloride (BDMDAC) on the degree of conversion, contact angle, ultimate tensile strength (UTS), microtensile bond strength (µTBS), cytotoxicity, antibacterial and bonding performance after artificial aging. A dental adhesive was doped with BDMDAC in the concentration range of 1–5 wt.%. For antibacterial assays, the BDMDAC compound was subject to planktonic cells of Streptococcus mutans. Then, after incorporation into the dental adhesive, an S. mutans biofilm model was used to grow 48 h-mature biofilms. The biofilms grown over the formulated materials were assessed by colony-forming unit (CFU) counting assay and fluorescence microscopy staining. In addition, the cytotoxicity was evaluated. Samples were subjected to 10,000 thermal cycles for aging and evaluated by UTS, µTBS, and CFU. Incorporating BDMDAC did not increase the cytotoxicity or change the physical properties when the mass fraction of the BDMDAC was 1–5 wt.%. The UTS of BDMDAC-doped adhesives was not impaired immediately or over time. A significant bacterial reduction was obtained for the mass fraction of the BDMDAC greater than 3 wt.%. However, the BDMDAC-doped adhesives did not offer an antibacterial effect after artificial aging. The overall results indicate that the BDMDAC strategy has the potential to control of microbial growth of cariogenic planktonic cells and biofilms. However, other new technological approaches are needed to overcome the deleterious effect of BDMDAC release over time such as those based on the principle of drug delivery systems whereby the BDMDAC is transported on microparticles or core shells, providing tangible benefits to oral health over time.

## 1. Introduction

Adhesion and subsequent growth of microorganisms on the tooth/material interface are major concerns preventing premature failures. Inside the mouth, tooth restorations are exposed to biofilm acid attacks over time. The degradation of adhesive by bacterial acids present over the interface tooth/restoration contributes to the recurrence of carious lesions around an existing restoration, namely secondary caries [1]. This process occurs mainly because of monomers’ cleavage due to the activity of water, saliva enzymes, and bacterial acids [2,3]. Consequently, improving adhesives’ properties to prevent biofilm formation on the margins of restorations is an exciting approach to assist restoration longevity [4].

The rapid development of biomaterials has forced equally rapid progress in dental materials [5]. As in the dental materials field, doping or incorporation is the intentional introduction of bioactive compounds, mostly antibacterial, into the formulation of the material to convey anti-biofilm properties to the final material [6]. The past decade has seen rapid development in nanotechnology in dentistry, with a notable advance in new dental materials for direct restorations, including dental adhesives [7]. These investigations have driven efforts on the effect of doping dental materials with inorganic metallic nanoparticles due to their potential antibacterial properties [8,9,10,11,12]. However, it should be noted that the nanoparticles have a much larger surface area to volume ratio due to their small size and, thus, highly increased activity [7]. Despite the many advantages, using metal and metal oxide nanoparticles is difficult. The main disadvantages include the increased cost associated with using nanotechnology, loss of antibacterial effect over time, complex synthesis through the multi-stage process, and the possible substantial color change of the final material [13].

In connection with the ongoing process of overcoming the challenges faced by nano-based strategies, great emphasis is put on finding new compounds with the potential to impart antibacterial effects to dental materials [14]. In the past decade, quaternary ammonium compounds have attracted considerable interest from academia and industry owing to their advantages over small molecular biocides, increased stability, low toxicity, facile synthesis, and the potential to reduce the broad spectra of oral pathogens [14]. Quaternary ammonium compounds are cationic surfactants that can disrupt the structure and integrity of cytoplasmic membranes and coagulating matrix constituents, causing enzyme denaturation [15] and inhibiting enzymes related to critical cellular functions such as respiration, energy transfer, ATP synthesis, and substrate oxidation [16]. Benzyldimethyldodecyl ammonium chloride (BDMDAC) is a benzylalkyldimethylethyl ammonium compound that can be bacteriostatic or bactericidal based on the concentration [17]. It is a cationic hydrophilic structure with a long alkyl chain of 12 carbons, which improves its antibacterial capacity. BDMDAC is a benzalkonium chloride component with antiseptic, detergent, and antibacterial properties [17]. It has been used in healthcare products as a preservative and antiseptic in eyewashes, nasal sprays, and injectable solutions [17]. However, there are no studies on doping BDMDAC to dental adhesives.

Therefore, this research aimed to develop the first dental adhesive doped with BDMDAC and study its effects on the degree of conversion, contact angle, ultimate tensile strength (UTS), microtensile bond strength (µTBS), cytotoxicity, and antibacterial and bonding performance after artificial aging. The objectives of the study were to: (1) synthesize BDMDAC-doped dental adhesive by incorporating BDMDAC in a range of 1–5 wt.% and investigate its effect on the mechanical and physicochemical properties before and after aging; (2) explore the antibacterial effect of BDMDAC-doped dental adhesive in a range of 1–5 wt.% before and after aging; (3) explore the cytotoxicity of BDMDAC-doped dental adhesive in a range of 1–5 wt.% on the studied concentrations.

## 2. Materials and Methods

### 2.1. Experimental Design

This is an in vitro experiment with six groups, as illustrated in Table 1.

**Figure 1:**
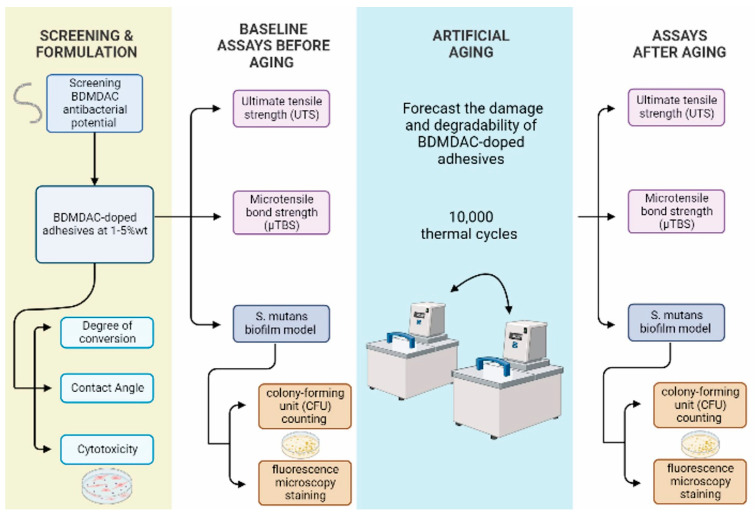
Displays the experimental flow chart procedures of screening and adhesives formulations, aging method, and assays before and after aging used in this study.

The outcome variables were degree of conversion, contact angle, ultimate tensile strength (UTS), microtensile bond strength (µTBS), cytotoxicity, and antibacterial and bonding performance after artificial aging. Experimental flow chart procedures of this study is presented in Figure 1.

### 2.2. Chemicals and Reagents

Materials purchased from Sigma-Aldrich (Saint Louis, MO, USA) and used without any purification: Camphorquinone; Ethyle 4-dimethylaminobenzoate; Butylated hydroxytoluene; and Benzyldimethldodecylammonium Chloride (BDMDAC). Monomers were purchased from Esstech (Essington, PA, USA): bisphenol A glycerolate dimethacrylate (BisGMA) and 2-hydroxyethyl methacrylate (HEMA); no further purification was made.

### 2.3. Determination of the Antibacterial Activity of Isolated BDMDAC Compound against S. mutans in Planktonic Cultures

The antibacterial activity of isolated BDMDAC compound against *S. mutans* in planktonic cultures was assessed to verify the potential antibacterial effect of this compound before incorporating it into the dental adhesive formulation. The bacterial suspension was adjusted with brain-heart infusion (BHI) broth to an initial optical density of 0.1 at 600 nm after being cultured for 24 h. Equal volumes of the diluted *S. mutans* liquid and BDMDAC (the final concentration is 10% (*w*/*v*), corresponding to 10 mg/mL) were mixed into 96-well plates. Then, we performed a dilution to achieve 7.5, 5, 3, 1.5, and 0.75% (*w*/*v*) of BDMDAC, followed by mixing solutions. Wells, without the addition of BDMDAC, served as negative growth controls. The 96-well plates were incubated at 37 °C for 24 h in an anaerobic incubator, and the inhibition of visible growth was observed. After, aliquots of each group were plated in BHI agar to observe the lowest concentration of BDMDAC at which there was no bacterial growth on the agar.

### 2.4. Formulation of Base Dental Adhesive Resins

Two methacrylate monomers were mixed in the following percentages to formulate the adhesive resins: 66.66 wt.% of bisphenol A glycerolate dimethacrylate (BisGMA) and 33.33 wt.% of 2-hyrdoxyethyl methacrylate (HEMA). Camphorquinone and ethyl 4-dimethylaminobenzoate were added at 1 mol% according to BisGMA and HEMA moles as photoinitiator/co-initiator systems. Butylated hydroxytoluene was added at 0.01 wt.% as a polymerization inhibitor [18]. Different concentrations of benzyldimethldodecylammonium chloride (BDMDAC) were selected following the minimum inhibitory concentration (MIC) and the minimum bactericidal concentration (MBC) results. BDMDAC was added to the adhesive resins’ formulation at 1, 2, 3, 4, and 5 wt.%. A group without BDMDAC was used as a control. The adhesives were mixed using a mechanical mixer (DAC 150 Speed mixer, Flacktec, Landrum, SC, USA) at 2800 rpm for 1 min.

### 2.5. Degree of Conversion

Prepared adhesives (*n* = 3) [19] were allotted directly on the ATR of FTIR crystal using a mold of polyvinylsiloxane to standardize sample thickness in 1 mm; each sample was covered by a polyester strip and photoactivated for 20 s using a light-cure unit with 1000 mW/cm^2^ placing the tip as close as possible to the sample surface [20]. Each sample was analyzed before and after the photoactivation using Opus 6.5 software and Blackman Harris 3-Term, OMNIC series at 4 cm^−1^ resolution. The degree of conversion was calculated using the peaks high at 1640 cm^−1^ and 1610 cm^−1^ following the below equation [20]:

$$\mathrm{DC}\;(\%)=100\;\times \;\left(\frac{peak\;height\;of\;cured\;aliphatic\;C=C/peak\;height\;of\;cured\;aromatic\;C=C}{peak\;height\;of\;uncured\;aliphatic\;C=C/peak\;height\;of\;uncured\;aromatic\;C=C}\right)$$

### 2.6. Ultimate Tensile Strength (UTS)

A metallic hourglass-shaped mold was used to prepare the samples (*n* = 5) [19] with the dimensions of (8 mm long, 2 mm wide, 1 mm thick, and a cross-sectional area of 1 mm^2^) [18]. Polyester strips were placed above and below each mold, and each sample was photoactivated for 20 s. After that, samples were measured using a digital caliper, then soaked in distilled water for 24 h at 37 °C. Finally, each sample was fixed in a metallic jig using cyanoacrylate resin, maintaining parallelism between the applied force and the sample’s long axis, in preparation for tension load in a universal testing machine (EZ-SX Series, Shimadzu, Kyoto, Japan) at 1 mm/min of crosshead speed. Later, samples were broken at the concentration area, values were obtained in newton (N) and divided by the concentration area revealing results in megapascal (MPa).

### 2.7. Microtensile Bond Strength (µTBS)

Extracted human second and third molars were used in this study after the university’s Institutional Board (IRB) approval (HP-00088564). First, each tooth was cut under running water at the cementoenamel junction to separate its roots [19]. Then, silicon carbide sandpaper (600-grit) was used to flatten the dentin and produce the smear layer for 30 s with distilled water irrigation [8,18].

Samples (*n* = 20) were then rinsed for 1 min and dried in preparation for bonding. Dentin was conditioned with 37% phosphoric acid for 15 s, then rinsed for 30 s and gently dried with absorbent paper to keep dentin moist. A primer (ScotchBond Multipurpose, 3M ESPE, Saint Paul, MN, USA) was applied actively for 20 s using a micro-brush, then the solvent evaporated via air drying. Next, adhesive resins were applied in two layers using a micro-brush and photoactivated for 20 s. A commercially available composite was used (Amelogen Plus A4, Ultradent Product, South Jordan, UT, USA) in two increments of 2 mm and photoactivated for 20 s in each layer. After that, samples were kept for 24 h in distilled water at 37 °C. Then, beams were cut in the size of 0.7 × 0.7 mm at the bonding area using (Isomet; #15HC blade 127 × 0.04 mm) under distilled water. Beam’s size was confirmed using an electronic caliper, fixed in a metallic jig via cyanoacrylate resin, and through the microtensile tester’s tensile strength (New Research Day, West Chicago, IL, USA) at 1 mm/min till a fracture occurs. Interface fractures’ values were recorded in newton (N), divided by the bonding area, and expressed in MPa [21]. Failures at the composite or dentin were excluded from the final tensile calculation as their values do not represent the dentin–adhesive interface. Fractured beams were classified according to their fracture location using a microscope (50X; VWR^®^ Stereo Zoom Binocular Microscope, Radnor, PA, USA) into adhesive (failure at the adhesive interface), mixed (at the adhesive interface with composite or dentin), cohesive in dentin, and cohesive in resin composite [22].

### 2.8. Contact Angle Assays

The contact angle was performed with water drops on the surface of the adhesive and with adhesive drops on the dentin surface. For the test with water, the sessile drop method was used to measure the static contact angle of water by dropping 10 µL on the surface of adhesive discs (*n* = 3) [23]. Three images were captured within 2 s of drop placement; the first image captured the droplet on a flat surface, creating the baseline. Later, the droplet edge and the gradient of the tangent of the droplet edge to the point where it meets the baseline were programmatically marked through goniometer software, and the resulting angle was measured from both sides. A single operator/observer was involved in this experiment [23].

For the test with the adhesives, a flat dentin surface was prepared [24], then the static contact angle was performed using the sessile drop method by placing a 10 µL of liquid adhesive using a micropipette (*n* = 3) [23]. A set of 3 images was captured within 2 s after placing the liquid adhesive on the dentin specimen; a contact angle goniometer was used for measurements. Again, a single operator was observing the measurements [25].

### 2.9. Sample Preparation for Microbiological Assays

Disc samples were prepared using a polyethylene mold with a diameter of 8 mm and a thickness of 1 mm [13,26]. Both disc surfaces were photoactivated for 20 s using a light-emitting diode with 1000 mW/cm^2^ (VALO Cordless, Ultradent Product, South Jordan, UT, USA). Later, samples were kept overnight in distilled water in an incubator at 37 °C, dried with absorbent paper, and sterilized with ethylene oxide gas (AnproleneAN 74i, Andersen, Haw River, NC, USA).

### 2.10. Streptococcus mutans Biofilm Model

An *S. mutans* biofilm model was used in this study. For that, the *Streptococcus mutans* strain (UA159) was cultured in BHI (Sigma-Aldrich) for 18 h in an incubator at 5% CO_2_ and 37 °C. The inoculum was prepared by adding 2 wt.% sucrose to the bacterial culture. Samples (*n* = 6) [19] were placed at the bottom of a 24-well plate. Then, 1.5 mL of the inoculum was added, and the plate was incubated for 24 h at 5% CO_2_ and 37 °C. Next, samples were transferred to a new 24-well plate containing 1.5 mL of fresh BHI broth supplemented with 2 wt.% of sucrose and incubated for 24 h at 5% CO_2_ and 37 °C [19].

### 2.11. Colony-Forming Unit (CFU) Counting Assay

CFU assay quantifies the total number of viable bacterial occurring in the 48 h biofilm. Biofilm was moved to a glass vial filled with 1 mL of cysteine peptide water (CPW) and collected by 10 s vortexing (BenchMixer Vortex Mixer, Benchmark Scientific, Inc., Sayreville, NJ, USA) 1 m sonication (CGOLDENWALL Ultrasonic Homogenizer, SPW Industrial, Laguna Hills, CA, USA) and 10 s vortexing. The bacterial suspension of each sample was serially diluted, and three drops of 10 µL from each dilution were plated onto BHI agar plates and incubated for 48 h at 5% CO_2_ and 37 °C for CFU analysis [27,28,29].

### 2.12. Fluorescence Microscopy Staining

The 2-day biofilm specimens were rinsed with phosphate-buffered saline (PBS) to eradicate non-adherent bacteria. Next, adhesive samples were stained via a live/dead bacterial kit (Molecular Probes, Eugene, OR, USA). A measurement of 2.5 µM of each SYTO 9 and propidium iodide was mixed and applied to the samples for 15 min. Propidium iodide stained the dead bacteria and emitted red fluorescence, and SYTO 9 stained live bacteria and emitted green fluorescence [30]. An inverted fluorescence microscope (Eclipse TE2000-S, Nikon, Melville, NY, USA) was used to image three stained disks per group, and five randomly picked views were taken for each sample yielding a total of 15 images per group.

### 2.13. Cytotoxicity Assay Using Human Gingival Fibroblasts

Adhesive disks (*n* = 3) [8] were prepared using a 4 mm diameter and 1 mm thickness mold, then photoactivated for 20 s on both sides. After that, they were sterilized using ethylene oxide, followed by seven days of degassing. Based on their relevance to dental adhesives, human gingival fibroblast (HGF, ScienCell, San Diego, CA, USA) was cultured in fibroblast medium (FM) supplemented with 2% fetal bovine serum, 100 IU/mL penicillin, and 100 IU/mL streptomycin. HGFs were inoculated into a 96-well plate at a density of 5000 cells/well in FM [31]. Each disk was immersed in a 4 mL medium for 24 h at 37 °C to obtain eluents [31,32]. As a result, the surface area/solution volume ratio was 0.63 cm^2^/mL, which was within the 0.5–6 cm^2^/mL range recommended by the International Organization for Standardization (ISO) [33,34]. The original extracts were diluted with fresh medium at 2-, 4-, 8-, 16-, 32-, 64-, and 128-fold [32], then the HGF were incubated in 100 µL of each sample’s original extract and their dilutions for 24 h. The HGFs in culture media without any extracts were used as a negative control. Following the incubation period, a 10 µL of cell counting kit-8 (CCK-8, Dojindo, Rockville, MD, USA) was added to each well and incubated for 2 h at 37 °C with 5% CO_2_ to evaluate the cell viability. Later, absorbance was measured (SpectraMAx M5) at 450 nm, representing the cellular dehydrogenase activity of live cells in culture media in percentages.

### 2.14. Artificial Aging by Thermocycling

A thermocycler (Odeme Thermocycling, OMC-250 L, Luzerna, Brazil) with a computer-controlled two-temperature cycler with distilled water baths of 5 and 55 °C was used. Each cycle consisted of 30 s immersion (dwell time) and 5 s transfer time [35,36]. After baseline measurements of antibacterial properties, microtensile, and ultimate tensile bond strength, samples were aged for 10,000 cycles to estimate a year of clinical service [37]. After the artificial aging, adhesive disks were sterilized with ethylene oxide gas, followed by seven days of degassing. Then the antibacterial tests, µTBS and UTS, were measured as previously described, and data were recorded as aged data.

### 2.15. Statistical Analysis

Data normality was analyzed using the Shapiro–Wilk test; one-way ANOVA and Tukey’s were conducted for multiple comparison tests. To compare data before and after aging, a *t*-test was performed. All tests were conducted at a significance level of 0.05 using SigmaPlot software version 12.0 (Systat Software, Inc., San Jose, CA, USA).

## 3. Results

**Figure 2:**
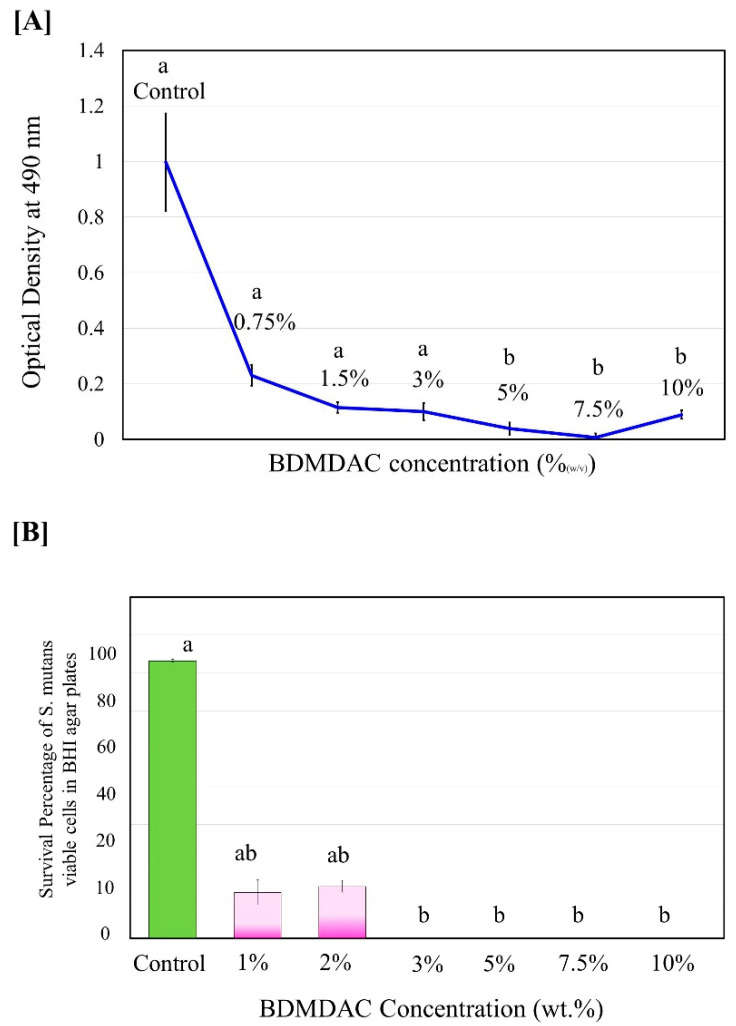
Antimicrobial potentiality of BDMDAC compound against *S. mutans* in planktonic cultures before being added to the dental adhesive formulation. (**A**) Growth curve of planktonic *S. mutans* subjected to contact with the BDMDAC concentrations of 0–10% (*w*/*v*). (**B**) Survival percentage of *S.mutans* viable planktonic cells growth in agar. Data are presented as the mean ± SD of three independent tests performed in triplicate. Means denoted by a different letter indicate significant differences between groups (*p* < 0.05).

The antibacterial activity of isolated BDMDAC compound against *S. mutans* in planktonic cultures was determined before being added to the dental adhesive formulation. Figure 2 shows the experimental results of the antibacterial activity assay expressed as the inhibition rate of the BDMDAC compound after incubation for 48 h on *S. mutans*. The complete death of *S. mutans* bacteria was observed when the concentration of BDMDAC compound was higher than 7.5 wt.% (Figure 2A), but the bacteriostatic rate was similar to that of 5 and 10 wt.%; the difference was not statistically significant (*p* ≥ 0.05). In addition, no bacterial growth in agar plates was observed for BDMDAC concentrations greater than 3 wt.% after 24 h (Figure 2B). There is a difference statistically significant for the concentrations at 1 and 2 wt.% (*p* ≤ 0.05).

**Figure 3:**
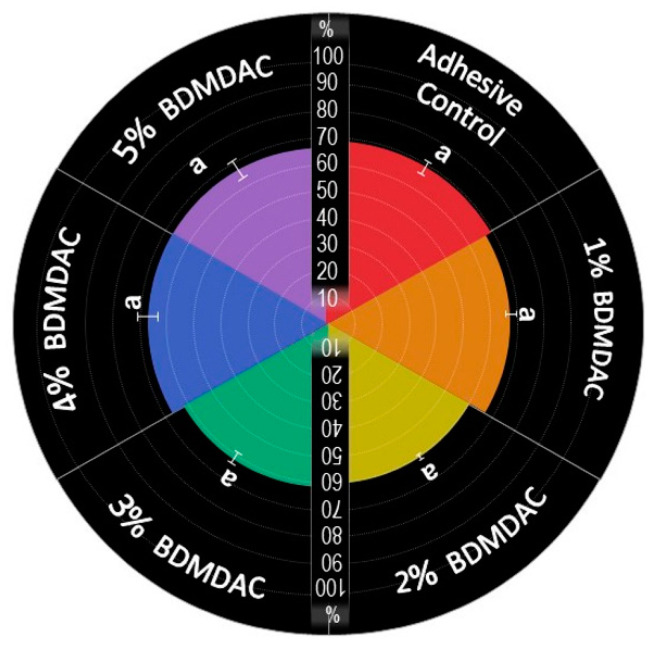
Percentage of the degree of conversion of experimental dental adhesives. The same letters indicate no statistically significant differences among the groups (*p* > 0.05).

The values of the degree of conversion are plotted in Figure 3. The results ranged from 60.84 (±1.62)% for BDMDAC 1 wt.% to 64.79 (±4.24)% for BDMDAC 5 wt.%, without statistically significant difference among them (*p* > 0.05). All groups reached values higher than 50% of conversion.

**Figure 4:**
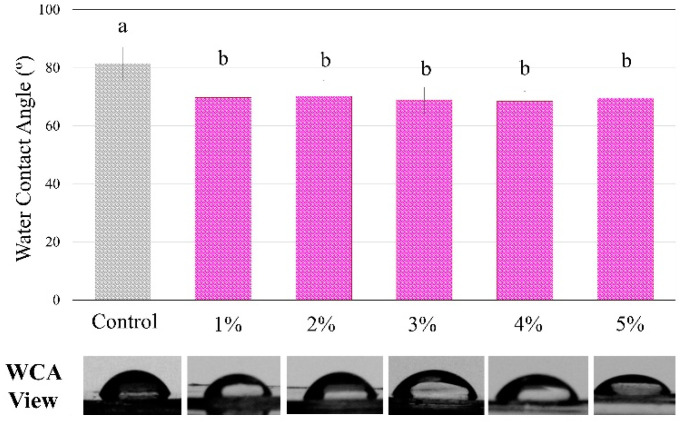
The degree of water contact angle and respective views of the control and BDMDAC- doped adhesive groups. Groups with the same letters are not statistically different (*p* > 0.05).

Figure 4 shows the analysis of the contact angle using water drops on the polymers showed statistically significant differences among groups, with a higher angle for the control compared to all BDMDAC groups (*p* < 0.05). However, there were no differences from 1 to 5 wt.% of BDMDAC (*p* > 0.05).

**Figure 5:**
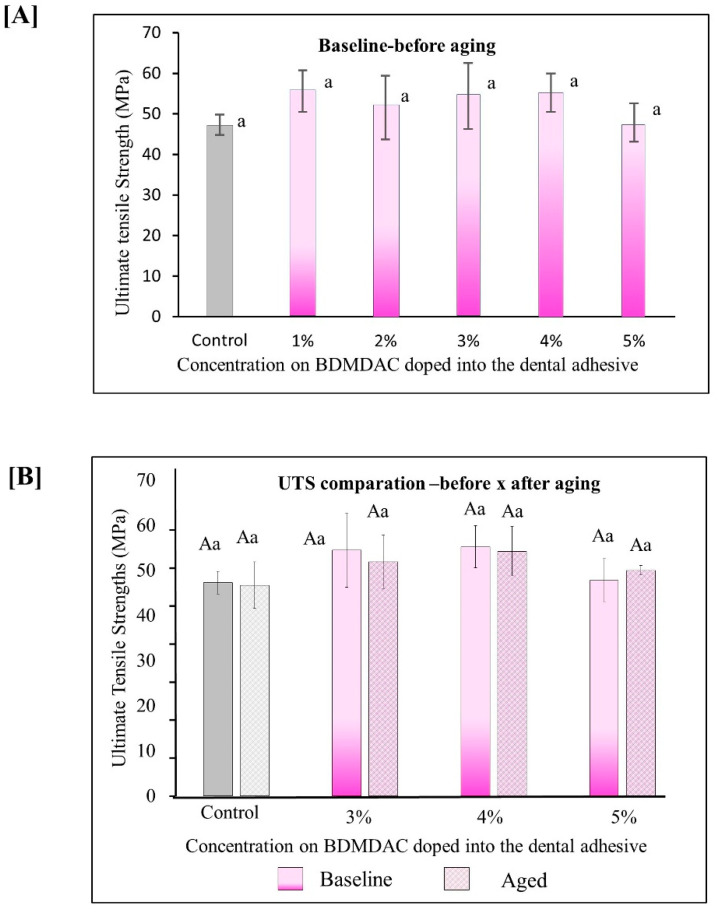
Results for ultimate tensile strength (UTS). Data are presented as the mean ± SD. (**A**) shows the immediate results of ultimate tensile strength for all the initial concentrated evaluated from 0 to 5 wt.%. BDMDAC. (**B**) shows the results for the adhesive formulations containing BDMDAC greater than 3 wt.% before and after aging. Means marked with the same lowercase letters do not differ significantly among the BDMDAC concentrations, and means marked with the same uppercase letters do not differ significantly between before and after aging; (*p* < 0.05).

Figure 5 describes the results for ultimate tensile strength (UTS). Figure 5A shows the immediate results of ultimate tensile strength for all the initial concentrated evaluated from 0 to 5 wt.% BDMDAC. The values ranged from 47.27 (±2.51) MPa for the control adhesive to 55.23 (±4.70) MPa for the group with 5 wt.% (*p* < 0.05). Figure 5B shows the results for the adhesive formulations containing BDMDAC greater than 3 wt.%. Please note that the formulations doped with BDMDAC at concentrations less than 3 wt.% were not subjected to artificial aging due to the lack of antibacterial potential of these formulations expressed in the antibacterial assays. After aging, the UTS of the adhesive formulations containing BDMDAC greater than 3 wt.% was not impaired. The values ranged from 46.70 (±5.14) MPa for control to 54.23 (±5.46) MPa for 5 wt.% (*p* < 0.05).

**Figure 6:**
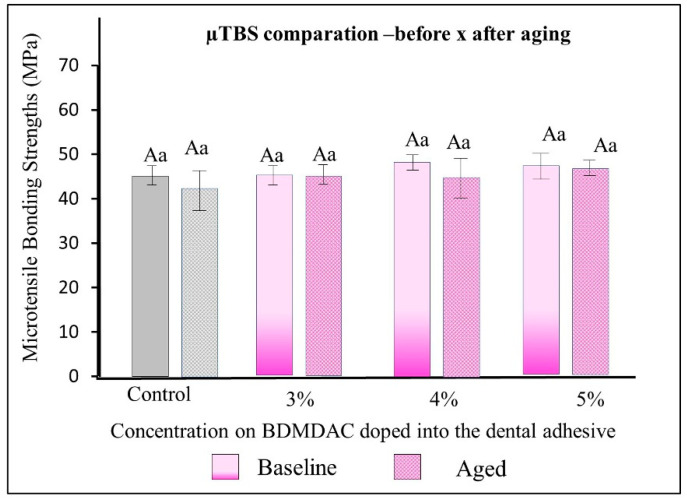
µTBS results of the dental adhesives immediately and after aging. There were no statistically significant differences among the formulated adhesives with 1 to 5 wt.% BDMDAC concentrations (comparative expressed by lowercase letters) or between before or after aging (comparative expressed by uppercase letters) (*p* > 0.05).

Figure 6 describes the µTBS results of the dental adhesives immediately and after aging. There were no statistically significant differences among the formulated adhesives with 1-to-5 wt.% BDMDAC concentrations (comparative expressed by lowercase letters) or between before or after aging (comparative expressed by uppercase letters) (*p* > 0.05).

**Figure 7:**
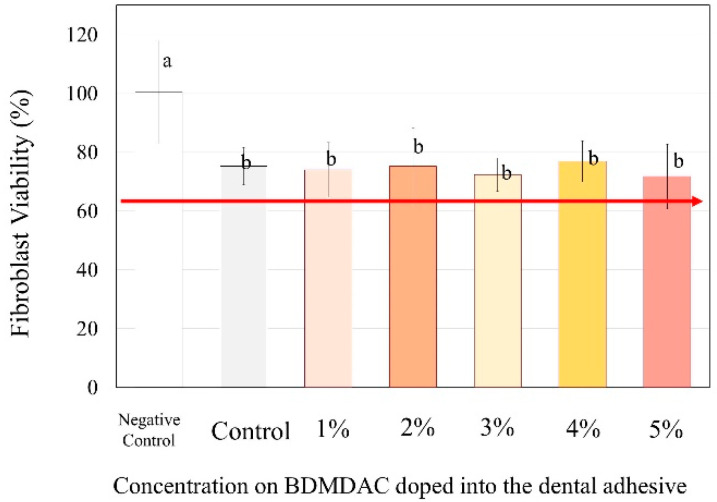
The response of the human gingival fibroblasts to contact with the BDMDAC-doped adhesives. Data are presented as the mean ± SD. Means marked with different lowercase letters differ significantly (*p* < 0.05).

Figure 7 presents the cytotoxicity assays performed to evaluate the biosafety of the different concentrations of BDMDAC incorporated with dental adhesives. The percentage of viable fibroblast cells exposed to adhesive control (BDMDAC not added) was approximately 80%, and the cell number differed significantly from that in the negative control (no adhesive added) (*p* < 0.05). However, the increased concentration of BDMDAC did not promote any more significant reduction in viability. Furthermore, there was no difference in cell viability among the groups in contact with the adhesives, but the viability differed in relation to the negative control (*p* < 0.05).

**Figure 8:**
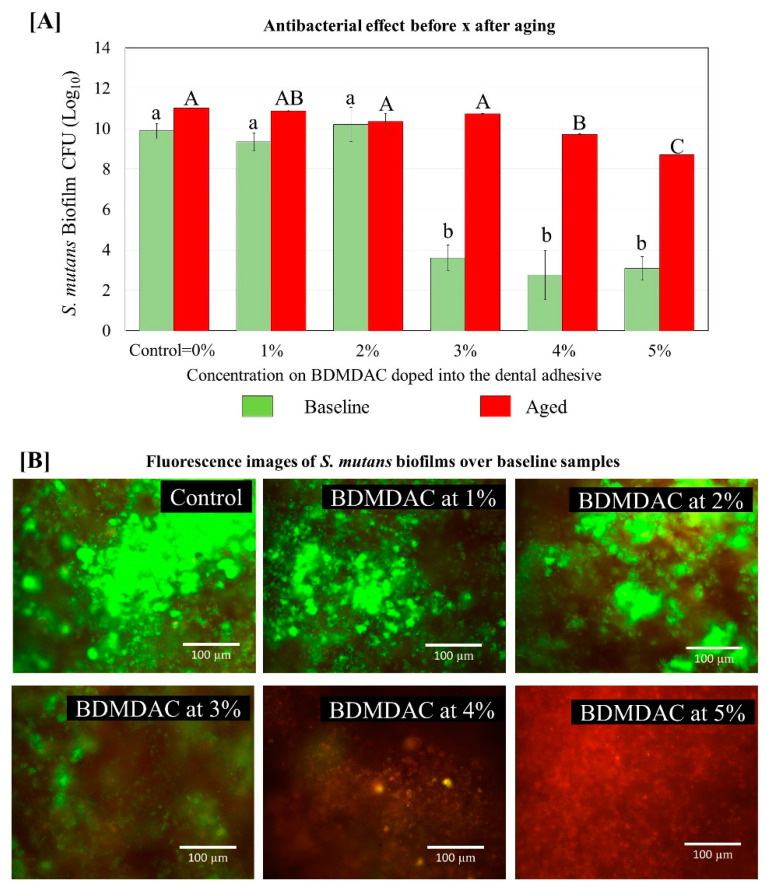
Colony-forming unit (CFU) counts for *S. mutans* biofilms grown over the BDMDAC-doped adhesives. (**A**) shows the CFU values for the biofilms grown over the samples immediately after being prepared (not subject to aging) versus the biofilm grown over the samples that were subjected to artificial aging promoted by 10,000 thermal cycles to simulate one year of service inside the mouth. Data are presented as the mean ± SD. Means marked with different lowercase letters differ significantly among the BDMDAC concentrations and means marked with different uppercase letters differ significantly between before and after aging; (*p* < 0.05). (**B**) Representative live/dead staining images of biofilms grown over baseline samples using fluorescence microscopy live bacteria were stained green, and compromised bacteria were stained red.

Figure 8 describes the antibacterial effect of the BDMDAC-doped adhesives against *S. mutans* biofilms before and after aging. Tests were performed at two different time points: immediately after the samples were prepared and after artificial aging simulating 1-year of service inside the mouth. The CFU analysis showed that an expressive bacterial reduction occurred on the biofilm grown over samples of adhesives containing BDMDAC at concentrations greater than 3 wt.% for the baseline testing (*p* < 0.05). However, after aging, these results were lost, and the performance of adhesives containing BDMDAC at concentrations greater than 3 wt.% was similar to the control (*p* > 0.05). The formulations containing 4 and 5% BDMDAC promoted a significant biofilm reduction, respectively; however, the performance was not even similar to the baseline results.

## 4. Discussion

The addition of antibacterial agents can modify the physicochemical properties of dental adhesives, which may jeopardize their application. In this study, we observed that BDMDAC addition did not change the degree of conversion and ultimate tensile strength up to 5 wt.%, with suitable µTBS with dentin immediately and after aging. Furthermore, the antibacterial activity was dose-dependent with the maintenance of the adhesive’s reliability for application without cytotoxic effects on human cells.

During the photoactivation of dental adhesives, free radicals are created and responsible for converting carbon double bonds (C=C) into single bonds (C-C) of methacrylate groups. When the conversion is proper, the polymer achieves reliable physicochemical properties, such as high ultimate tensile strength and hardness and low sorption and solubility. Previous reports in the literature have addressed the incorporation of quaternary ammonium monomers into dental adhesives and investigated the degree of conversion [38,39]. Liang et al. [40] demonstrated that quaternary ammonium monomers negatively affect the photopolymerization of the relevant dental resin within the concentration range of 5–20 wt.%.

As a limiting factor, incorporating antibacterial agents into the organic matrix can decrease the conversion degree by influencing the polymer’s color or viscosity. BDMDAC is a quaternary ammonium-derivative molecule that does not present C=C on its structure and will not covalently bond with the monomers. Occasionally, the polymerization kinetics is modified by incorporating such molecules, decreasing the resin’s viscosity, increasing monomer chain mobility, and increasing the maximum polymerization rate. However, as previously observed with a quaternary ammonium-derivative molecule without methacrylate groups, the degree of conversion did not change, and all groups presented high values similar to commercial adhesives [38,39,40,41].

The degree of conversion provides critical data about the chemistry of the developed material [42]. However, this test does not show how the polymeric network is formed. Materials can achieve a high degree of conversion but achieve a more linear polymeric network with lower crosslink density [43]. The added agent can act as a plasticizer, pulling the chains apart. Therefore, the mechanical properties of the polymer must be tested, such as ultimate tensile strength. In the present research, besides no differences in the degree of conversion, the mechanical properties of the adhesives were also not influenced up to 5 wt.% addition of BDMDAC.

Despite good immediate results, the ability to maintain the adhesion must be analyzed over time, especially when dentin is involved in dental restoration. During aging, the cured adhesive is prone to swell and suffers hydrolysis and degradation [44]. Moreover, the collagen fibrils are also attacked by water and active MMP, cleaving the collagen structure [45]. Nevertheless, as observed in the degree of conversion and contact angle of the adhesives, the doping of BDMDAC has not impaired their performance since, overall, the BDMDAC-doped materials performed similarly to control regarding the physical–chemical properties.

The rationale for such results is based on the bacterial inhibition mechanism of quaternary ammonium compounds, in which the positively charged quaternary amine interacts with the negatively charged bacterial cell membrane, causing cytoplasmic leakage and bacterial death [46]. We chose to use BDMDAC due to its long alkyl chain with sixteen carbons, increasing the lipophilicity of the molecule and the chances of bacterial interaction. In addition, BDMDAC is a quaternary ammonium-derivative molecule without methacrylate groups. Therefore, the polymeric network’s part of the BDMDAC entrapped may be leached over time. Few studies analyze the antibacterial activity after aging [47,48]. However, long-term evaluation is critical as a preclinical assessment of the clinical performance during biomaterials development [49], especially when the antibacterial agent cannot chemically bond with the matrix. Therefore, we tested the adhesives samples immediately and after aging in artificial saliva. As expected, all groups with BDMDAC showed lower antibacterial activity after aging. This study’s limitation is that we did not quantify the antibacterial agent leached over time. However, it is possible to state that some part was lost over time.

Interestingly, the leaching did not affect the bonding to dentin, as they maintained a high µTBS over time, with no statistical differences for the immediate analysis. Similar results were observed in the literature. Pupo et al. [41] investigated the resin–dentin bond strength (μTBS), degree of conversion (DC), and antibacterial potential of commercial adhesive modified with quaternary ammonium methacrylate polymer (QAMP) at 5% and compared it with Clearfil™ Protect Bond. No statistically significant difference in μTBS was observed between Clearfil™ SE Bond containing 5% QAMP and control immediately and after 6 and 12 months of water storage. However, Clearfil™ Protect Bond showed a significant reduction of μTBS after 12 months of storage. In addition, QAMP provided no significant change in DC after incorporating it into Clearfil™ SE Bond.

Future studies could be addressed to synthesize drug delivery systems with BDMDAC able to control its release rate. In addition, Nanotubes, nanocapsules, microspheres, and core shells have increasingly emerged in dentistry and are encouraged to overcome the issue related to the fast leaching of antibacterial agents without methacrylate groups.

Finally, the adhesives were tested for possible cytotoxic effects on human cells. Incorporating antibacterial agents into dental materials should not affect their mechanical properties. Furthermore, the antibacterial agents should not generate toxicity in the surrounding tissues. Here we had no statistical differences among the experimental adhesives. However, in a previous study [50], myristyl trimethyl ammonium bromide was incorporated into a dental resin up to 2 wt.% and showed cytotoxic effects against human keratinocytes (HaCaT) compared to the control group and 0.5 wt.%, achieving percentages of cell viability below that recommended by the International Organization for Standardization (ISO), which states that biomaterials need to show at least 70% of cells viability not to be considered cytotoxic.

## 5. Conclusions

The present study combined a not-yet-explored quaternary ammonium-derivative molecule into a dental resin, which attained effective antibacterial activity against a *Streptococcus mutans* biofilm. The results showed that: (1) BDMDAC did not influence the physicochemical properties of the adhesives immediately or over time; (2) The lower the concentration of BDMDAC, the greater the antibacterial effect. However, this effect was lower after sample aging; (3) BDMDAC at a maximum concentration of 5 wt.% had no detrimental effect on the viability of human cells. In conclusion, BDMDAC is a promising antibacterial agent for dental applications, and future studies should be enrolled to keep this agent in the polymeric network or control its release over time.

## Figures and Tables

**Table 1:** Description of the dental adhesive formulations investigated in this study.

Group #	BDMDAC Concentration
Group 1 = Control	Adhesive + 0 wt.% BDMDAC
Group 2	Adhesive + 1 wt.% BDMDAC
Group 3	Adhesive + 3 wt.% BDMDAC
Group 4	Adhesive + 4 wt.% BDMDAC
Group 5	Adhesive + 5 wt.% BDMDAC

## Data Availability

Data are contained within the article.
